# Supplementary material for: Associations between neighbourhood characteristics and participation in a population-based organised prostate cancer testing (OPT) programme: A register-based study of 50-year-old men
Source: PLoS One. 2025 Apr 29;20(4):e0322643. doi: 10.1371/journal.pone.0322643 (PMC12040343; doi:10.1371/journal.pone.0322643)
Supplement: S1 Text — Text file containing the equation for spatial modelling, supplementary tables 1–9 and a text about population distribution across neighbourhoods. (DOCX) [file pone.0322643.s001.docx]

# Supplement

## Spatial model

For the i-th residential neighbourhood (i = 1, 2, …, 992), the logit-transformed participation rates were modelled as

$$\mathrm{logit}\left( p_{i} \right)=\alpha+b_{i}$$

where α is the common intercept which represents the baseline participation rate, with a combination of a conditional autoregressive structure ($u_{i}$), to capture local dependency and independent and identically distributed Gaussian random effects ($v_{i}$) to account for the similarities of all the areas in the whole study region:

$$b_{i}= v_{i}+u_{i}.$$

This model provides both local and global smoothing on the underlying participation rate p_i_. We specified a minimally informative prior on the logs of both the unstructured and structured precision, logGamma(1, 0.0005).

*Demographic Statistical Areas (DeSOs): correlations and characteristics*

The strong correlation between deprivation index and income has been previously established (26) and further validated by our results.

| **Supplementary table 1. Deprivation (an index of multiple deprivation) * Proportion of inhabitants with low economic standard Crosstabulation** | | | | | | | | |
| --- | --- | --- | --- | --- | --- | --- | --- | --- |
| Spearman’s correlation r_s_ = 0.94 | | | Proportion of inhabitants with low economic standard | | | | | Total |
|  |  |  | Q1 | Q2 | Q3 | Q4 | Q5 |  |
| Deprivation (an index of multiple deprivation) | Q1 | Count | 5,113 | 541 | 0 | 0 | 0 | 5,654 |
|  |  | % within Deprivation (an index of multiple deprivation) | 90.4% | 9.6% | 0.0% | 0.0% | 0.0% | 100.0% |
|  | Q2 | Count | 415 | 2,836 | 773 | 11 | 0 | 4,035 |
|  |  | % within Deprivation (an index of multiple deprivation) | 10.3% | 70.3% | 19.2% | 0.3% | 0.0% | 100.0% |
|  | Q3 | Count | 49 | 912 | 2,505 | 304 | 0 | 3,770 |
|  |  | % within Deprivation (an index of multiple deprivation) | 1.3% | 24.2% | 66.4% | 8.1% | 0.0% | 100.0% |
|  | Q4 | Count | 0 | 74 | 893 | 2,548 | 258 | 3,773 |
|  |  | % within Deprivation (an index of multiple deprivation) | 0.0% | 2.0% | 23.7% | 67.5% | 6.8% | 100.0% |
|  | Q5 | Count | 0 | 0 | 0 | 714 | 3,002 | 3,716 |
|  |  | % within Deprivation (an index of multiple deprivation) | 0.0% | 0.0% | 0.0% | 19.2% | 80.8% | 100.0% |
| Total | | Count | 5,577 | 4,363 | 4,171 | 3,577 | 3,260 | 20,948 |
|  |  | % within Deprivation (an index of multiple deprivation) | 26.6% | 20.8% | 19.9% | 17.1% | 15.6% | 100.0% |

| **Supplementary table 2.** **Deprivation (an index of multiple deprivation) * Proportion of inhabitants aged 25-64 years with <=12 years in school Crosstabulation** | | | | | | | | |
| --- | --- | --- | --- | --- | --- | --- | --- | --- |
| Spearman’s correlation r_s_ = 0.49 | | | Proportion of inhabitants aged 25-64 years with <=12 years in school | | | | | Total |
|  |  |  | Q1 | Q2 | Q3 | Q4 | Q5 |  |
| Deprivation (an index of multiple deprivation) | Q1 | Count | 2,244 | 2,275 | 915 | 208 | 12 | 5,654 |
|  |  | % within Deprivation (an index of multiple deprivation) | 39.7% | 40.2% | 16.2% | 3.7% | 0.2% | 100.0% |
|  | Q2 | Count | 563 | 814 | 1,179 | 1,282 | 197 | 4,035 |
|  |  | % within Deprivation (an index of multiple deprivation) | 14.0% | 20.2% | 29.2% | 31.8% | 4.9% | 100.0% |
|  | Q3 | Count | 625 | 756 | 956 | 835 | 598 | 3,770 |
|  |  | % within Deprivation (an index of multiple deprivation) | 16.6% | 20.1% | 25.4% | 22.1% | 15.9% | 100.0% |
|  | Q4 | Count | 711 | 631 | 672 | 716 | 1,043 | 3,773 |
|  |  | % within Deprivation (an index of multiple deprivation) | 18.8% | 16.7% | 17.8% | 19.0% | 27.6% | 100.0% |
|  | Q5 | Count | 196 | 299 | 668 | 1,099 | 1,454 | 3,716 |
|  |  | % within Deprivation (an index of multiple deprivation) | 5.3% | 8.0% | 18.0% | 29.6% | 39.1% | 100.0% |
| Total | | Count | 4,339 | 4,775 | 4,390 | 4,140 | 3,304 | 20,948 |
|  |  | % within Deprivation (an index of multiple deprivation) | 20.7% | 22.8% | 21.0% | 19.8% | 15.8% | 100.0% |

| **Supplementary table 3.** **Deprivation (an index of multiple deprivation) * Proportion of non-Western immigrants Crosstabulation** | | | | | | | | |
| --- | --- | --- | --- | --- | --- | --- | --- | --- |
| Spearman’s correlation r_s_ = 0.56 | | | Proportion of non-Western immigrants | | | | | Total |
|  |  |  | Q1 | Q2 | Q3 | Q4 | Q5 |  |
| Deprivation (an index of multiple deprivation) | Q1 | Count | 1,475 | 2,214 | 1,357 | 478 | 130 | 5,654 |
|  |  | % within Deprivation (an index of multiple deprivation) | 26.1% | 39.2% | 24.0% | 8.5% | 2.3% | 100.0% |
|  | Q2 | Count | 917 | 1,219 | 1,017 | 582 | 300 | 4,035 |
|  |  | % within Deprivation (an index of multiple deprivation) | 22.7% | 30.2% | 25.2% | 14.4% | 7.4% | 100.0% |
|  | Q3 | Count | 686 | 690 | 885 | 1,018 | 491 | 3,770 |
|  |  | % within Deprivation (an index of multiple deprivation) | 18.2% | 18.3% | 23.5% | 27.0% | 13.0% | 100.0% |
|  | Q4 | Count | 240 | 622 | 1,136 | 1,118 | 657 | 3,773 |
|  |  | % within Deprivation (an index of multiple deprivation) | 6.4% | 16.5% | 30.1% | 29.6% | 17.4% | 100.0% |
|  | Q5 | Count | 46 | 85 | 302 | 807 | 2,476 | 3,716 |
|  |  | % within Deprivation (an index of multiple deprivation) | 1.2% | 2.3% | 8.1% | 21.7% | 66.6% | 100.0% |
| Total | | Count | 3,364 | 4,830 | 4,697 | 4,003 | 4,054 | 20,948 |
|  |  | % within Deprivation (an index of multiple deprivation) | 16.1% | 23.1% | 22.4% | 19.1% | 19.4% | 100.0% |

| **Supplementary table 4.** **Deprivation (an index of multiple deprivation) * Degree of urbanisation Crosstabulation** | | | | | | |
| --- | --- | --- | --- | --- | --- | --- |
| Spearman’s correlation r_s_ = 0.11 | | | Degree of urbanisation | | | Total |
|  |  |  | Rural | Semi-urban | Urban |  |
| Deprivation (an index of multiple deprivation) | Q1 | Count | 548 | 335 | 4,771 | 5,654 |
|  |  | % within Deprivation (an index of multiple deprivation) | 9.7% | 5.9% | 84.4% | 100.0% |
|  | Q2 | Count | 1,623 | 367 | 2,045 | 4,035 |
|  |  | % within Deprivation (an index of multiple deprivation) | 40.2% | 9.1% | 50.7% | 100.0% |
|  | Q3 | Count | 1,047 | 313 | 2,410 | 3,770 |
|  |  | % within Deprivation (an index of multiple deprivation) | 27.8% | 8.3% | 63.9% | 100.0% |
|  | Q4 | Count | 444 | 312 | 3,017 | 3,773 |
|  |  | % within Deprivation (an index of multiple deprivation) | 11.8% | 8.3% | 80.0% | 100.0% |
|  | Q5 | Count | 41 | 119 | 3,556 | 3,716 |
|  |  | % within Deprivation (an index of multiple deprivation) | 1.1% | 3.2% | 95.7% | 100.0% |
| Total | | Count | 3,703 | 1,446 | 15,799 | 20,948 |
|  |  | % within Deprivation (an index of multiple deprivation) | 17.7% | 6.9% | 75.4% | 100.0% |

*Population distribution across neighbourhoods*

To better understand the population distribution across neighbourhood areas with varying characteristics, we utilised individual-level data obtained from Statistics Sweden, covering all men in Region Västra Götaland in 2020. The dataset included 56,362 men aged 50-54 years, with information on disposable income and country of birth. Economic status was categorized as low (disposable income below the lowest quartile of Swedish households, cutoff ≤20,698 USD/year), medium (middle two quartiles, 20,699 - 39,037 USD/year), and high (upper quartile, ≥39,038 USD/year). Country of birth were analysed as proportion of non-western immigrants. Non-Western countries were defined as Eastern Europe, Asia, Africa and South America.

By integrating these data with the DeSO dataset, we analysed the distribution of this population across the DeSOs in relation to the examined socioeconomic factors according to each quintile and level of urbanisation. The findings indicate that 14,681 (26.1%) of the 56,362 men aged 50-54 in Region Västra Götaland were categorized in quintile one for deprivation, while 11,084 (19.7%) were assigned to quintile two, and the distribution continued similarly for subsequent quintiles. This analysis suggests that the population from Västra Götaland closely aligns with the national quintile distribution, with a possible overrepresentation of men living in areas with low deprivation and high economical standard. If the Region Västra Götaland population completely matched the national population, the valid percentage for each quintile would be 20%. 41,747 (74.1%) of the men were living in rural areas.

| **Supplementary table 5.** **DeSO – deprivation index** | | | | | |
| --- | --- | --- | --- | --- | --- |
|  | | Frequency | Percent | Valid Percent | Cumulative Percent |
| Valid | Q1 | 14,681 | 26.0 | 26.1 | 26.1 |
|  | Q2 | 11,084 | 19.7 | 19.7 | 45.8 |
|  | Q3 | 10,410 | 18.5 | 18.5 | 64.4 |
|  | Q4 | 10,264 | 18.2 | 18.3 | 82.6 |
|  | Q5 | 9,766 | 17.3 | 17.4 | 100.0 |
|  | Total | 56,205 | 99.7 | 100.0 |  |
| Missing | System | 157 | 0.3 |  |  |
| Total | | 56,362 | 100.0 |  |  |

| **Supplementary table 6.** **DeSO – low economical standard** | | | | | |
| --- | --- | --- | --- | --- | --- |
|  | | Frequency | Percent | Valid Percent | Cumulative Percent |
| Valid | Q1 | 14,519 | 25.8 | 25.8 | 25.8 |
|  | Q2 | 11,917 | 21.1 | 21.2 | 47.0 |
|  | Q3 | 11,485 | 20.4 | 20.4 | 67.5 |
|  | Q4 | 9,801 | 17.4 | 17.4 | 84.9 |
|  | Q5 | 8,483 | 15.1 | 15.1 | 100.0 |
|  | Total | 56,205 | 99.7 | 100.0 |  |
| Missing | System | 157 | 0.3 |  |  |
| Total | | 56,362 | 100.0 |  |  |

| **Supplementary table 7.** **DeSO – inhabitants aged 25 – 64 with ≤ 12 years in school** | | | | | |
| --- | --- | --- | --- | --- | --- |
|  | | Frequency | Percent | Valid Percent | Cumulative Percent |
| Valid | Q1 | 11,196 | 19.9 | 19.9 | 19.9 |
|  | Q2 | 12,584 | 22.3 | 22.4 | 42.3 |
|  | Q3 | 12,053 | 21.4 | 21.4 | 63.8 |
|  | Q4 | 11,484 | 20.4 | 20.4 | 84.2 |
|  | Q5 | 8,888 | 15.8 | 15.8 | 100.0 |
|  | Total | 56,205 | 99.7 | 100.0 |  |
| Missing | System | 157 | 0.3 |  |  |
| Total | | 56,362 | 100.0 |  |  |

| **Supplementary table 8.** **DeSO – proportions of non-western immigrants** | | | | | |
| --- | --- | --- | --- | --- | --- |
|  | | Frequency | Percent | Valid Percent | Cumulative Percent |
| Valid | Q1 | 9,488 | 16.8 | 16.9 | 16.9 |
|  | Q2 | 13,052 | 23.2 | 23.2 | 40.1 |
|  | Q3 | 12,283 | 21.8 | 21.9 | 62.0 |
|  | Q4 | 10,938 | 19.4 | 19.5 | 81.4 |
|  | Q5 | 10,444 | 18.5 | 18.6 | 100.0 |
|  | Total | 56,205 | 99.7 | 100.0 |  |
| Missing | System | 157 | 0.3 |  |  |
| Total | | 56,362 | 100.0 |  |  |

| **Supplementary table 9.** **DeSO - Urbanisation** | | | | | |
| --- | --- | --- | --- | --- | --- |
|  | | Frequency | Percent | Valid Percent | Cumulative Percent |
| Valid | Rural | 10,723 | 19.0 | 19.1 | 19.1 |
|  | Semi-urban | 3,735 | 6.6 | 6.6 | 25.7 |
|  | Urban | 41,747 | 74.1 | 74.3 | 100.0 |
|  | Total | 56,205 | 99.7 | 100.0 |  |
| Missing | System | 157 | 0.3 |  |  |
| Total | | 56,362 | 100.0 |  |  |
