# Supplementary material for: Associations between neighbourhood characteristics and participation in a population-based organised prostate cancer testing (OPT) programme: A register-based study of 50-year-old men
Source: PLoS One. 2025 Apr 29;20(4):e0322643. doi: 10.1371/journal.pone.0322643 (PMC12040343; doi:10.1371/journal.pone.0322643)
Supplement: Attachment — Submitted filename: PLOS One.docx [file pone.0322643.s002.docx]

Row 86 –when possible morbidity

Please, definition of deprivation

Part “Population distribution across neighbourhoods” from the Suppplement should be included in methodology but I suggest several changes:

To better understand the population distribution across neighbourhood areas with varying characteristics, we utilised individual-level data obtained from Statistics Sweden, covering all men in Region Västra Götaland in 2020. The dataset included 56,362 men aged 50-54 years, with information on disposable income and country of birth. Economic status was categorized as low (disposable income below the lowest quartile of Swedish households), medium (middle two quartiles), and high (upper quartile)

By integrating this data with the DeSO dataset, we analysed the distribution of this population across the DeSOs in relation to the examined socioeconomic factors for each quintile and levels of urbanisation. The findings indicate that 14,681 (26.1%) of the 56,362 men aged 50-54 in Region Västra Götaland were categorized in quintile one for deprivation, while 11,084 (19.7%) were assigned to quintile two, and the distribution continued similarly for subsequent quintiles. This analysis suggests that the population from Västra Götaland closely aligns with the national quintile distribution, with a possible overrepresentation of men living in areas with low deprivation and high economical standard. If the Region Västra Götaland population completely matched the national population, the valid percentage for each quintile would be 20%. 41,747 (74.1%) of the men were living in rural areas.
