# Supplementary material for: Associations between neighbourhood characteristics and participation in a population-based organised prostate cancer testing (OPT) programme: A register-based study of 50-year-old men
Source: PLoS One. 2025 Apr 29;20(4):e0322643. doi: 10.1371/journal.pone.0322643 (PMC12040343; doi:10.1371/journal.pone.0322643)
Supplement: Attachment — Submitted filename: Response to reviewers EJ250313.docx [file pone.0322643.s004.docx]

**Lorenzo Righi**

**Academic editor**

PLOS ONE

11^th^ Mars 2025

Dear Dr Righi / Associate Editors,

Thank you for considering our manuscript for publication. Your and the reviewers’ comments have been valuable for improving the manuscript. We hope that you will find the revised version suitable for publication.

Our responses to the comments are listed below.

**Response to the reviewers’ comments:**

Reviewer #1:1: Why just include men who are 50 years? Was it to just include men who are first invited by the programme to participate (i.e their first screening test). What about men aged >50 years? Other screening programmes have found age related differences in screening uptake. I may have missed this in the paper but are men screened on an annual basis? Or what is the screening interval?

Response 1:1: We realise that this was not made clear and added, “Because of the decision to start with 50-year-old men and the biannual invitation system of the OPT, only 50-year-old men were invited during this period.”, in the Methods section under subheading study population, row 112.

Reviewer #1:2: Comparisons with established cancer screening programmes' – maybe add that the relative newness of the the implementation of the OPE programmes may be a factor for lower participation compared to other programmes

Response 1:2: We agree that this is relevant and added, “The newness of the OPT concept may contribute to a lower participation rate.” (Discussion, row 280).

Reviewer #1:3: In Sweden, neighbourhood income is more strongly associated with OPT participation than neighbourhood education level and proportion of non-Western immigrants in the neighbourhood' - can you give a possible rationale for this finding

Response 1:3: We appreciate this comment as these are important aspects for the interpretation of our results. There may be several reasons why income is more strongly associated with participation, but we are not aware of any studies that could shed light on them. Rather than speculating, we added further one reference to a study with similar findings: “… as well as with a report of single deprivation indicators versus an index of deprivation on all cause mortality (26.)”, on row 254.

Reviewer #1:4: I think it is also important to point out that what might work in terms of interventions in one screening programme (e.g. cervical) may not work in other programmes (e.g. prostate) as you are dealing with different populations

Response 1:4: We agree and rephrased to “Home-based testing has proven effective in cervical cancer prevention (ref), and home-based PSA testing is currently being evaluated in Swedish regions with a high proportion of men with a long distance to a testing facility. Interventions that are effective for raising participation in one screening programme may, however, not work in others.”, on rows 261 - 265.

Reviewer #2:1: Please, provide the definition of deprivation related to your paper and also criteria for economic status in USD.

Response 2:1: We appreciate this comment and agree that the definition needed clarification and that status in USD would provide valuable information and enable easier comparisons between settings. We therefore added “Disposable income per consumption unit is a metric commonly employed by Statistics Sweden to assess economic standards, representing the total household income available after taxes, adjusted for the number of individuals in the household. Low economic standard was defined as the lower quartile of disposable income per consumption unit among all Swedish households (cutoff at 20,698 USD/year in 2020), row 137 – 142.

Reviewer #2:2: Part “Population distribution across neighbourhoods” from the Suppplement should be included in methodology but I suggest several changes:

Response 2:2: We added “Correlation between neighbourhood characteristics was explored using cross tabulation shown in supplementary tables 1 - 4. We also analysed the population distribution across neighbourhoods in supplementary tables 5 – 9.”, in Methods, row 154.
For suggested changes, see below.

Reviewer #2:3: Please provide more information about the importance of “country of birth” related to this sentence: "The dataset included 56,362 men aged 50-54 years, with information on disposable income and country of birth."

Response 2:3: We appreciate the comment. To clarify that country of birth was defined the same way here as in the other population**,** we added at the end of that paragraph in the supplements**:** “Country of birth were analysed as proportion of non-western immigrants. Non-Western countries were defined as Eastern Europe, Asia, Africa and South America.”

Reviewer #2:4: Please, provide the criteria for economic status in USD related to this sentence: "Economic status was categorized as low (disposable income below the lowest quartile of Swedish households), medium (middle two quartiles), and high (upper quartile)"

Response 2:4: We agree that economic status in USD would be valuable here and added “cutoff ≤20,698 USD/year), medium (middle two quartiles, 20,699 - 39,037 USD/year), and high (upper quartile, ≥39,038 USD/year).”, in that part of the supplements.

Reviewer #2:5: "By integrating this data with the DeSO dataset, we analysed the distribution of this population across the DeSOs in relation to the examined socioeconomic factors for each quintile and levels of urbanisation. ”Suggestion for changes: in relation to the examined socioeconomic factors according to each quintile and level of urbanization

Response 2:5: We are grateful for this suggestion and rephrased as suggested.

**Editorial comments**

We have adapted the manuscript to PLOS ONE's style requirements and:

- Added the requested information about funders, in the cover letter as requested,
- Added the requested information in declared conflicts of interest, in the cover letter as requested,
- Removed the “Title page” and “statements and declarations”, and only mention our ethical statement in the methods section,
- The figures were made with open data from Stastistics Sweden and the authors are the owners of the figures. Statistics Sweden states that unaltered statistics used from their databases should be referred to Statistics Sweden but that altered statistics, such as our maps, should not be referred. For more information, see https://www.scb.se/en/services/open-data-api/open-geodata/
- added captions for supporting information,
- checked the reference list,

Sincerely yours

On behalf of all the authors

***Emil Järbur***

PhD student and urologist

University of Gothenburg, Sweden
